# A checklist of marine bryozoan taxa in Scottish sea regions

**DOI:** 10.3897/zookeys.787.24647

**Published:** 2018-10-03

**Authors:** Sally Rouse, Jennifer Loxton, Mary E. Spencer Jones, Joanne S. Porter

**Affiliations:** 1 Scottish Association for Marine Science, Oban, Argyll, PA37 1QA, UK Scottish Association for Marine Science Oban United Kingdom; 2 School of Geosciences, University of Edinburgh, Edinburgh, EH9 3FE, UK University of Edinburgh Edinburgh United Kingdom; 3 Department of Life Sciences, Natural History Museum, Cromwell Road, London, SW7 5BD, UK Natural History Museum London United Kingdom; 4 International Centre for Island Technology, Heriot Watt University, Orkney, Old Academy, Back Road, Stromness, KW16 3DA, UK Heriot Watt University Stromness United Kingdom

**Keywords:** Bryozoa, distribution, non-indigenous species, Scotland

## Abstract

Contemporary and historical bryozoan records were compiled to provide a comprehensive checklist of species in Scottish waters. The checklist comprises 218 species in 58 families, with representatives from each of the extant bryozoan orders. The fauna was relatively sparse compared to other regions for which bryozoan checklists were available e.g. New Zealand and Australia. Six non-indigenous bryozoan species from the Scottish seas region were included in the checklist. Baseline information on species distributions, such as that presented in this checklist, can be used to monitor and manage the impact of human activities on the marine environment, and ultimately preserve marine biodiversity.

## Introduction

The phylum Bryozoa comprises approximately 6000 known/described extant species of filter feeding invertebrates that predominantly occur in the marine environment (Gordon and Costello 2016). There are three classes and four orders of extant bryozoans (class Gymnolaemata, orders Cheilostomatida and Ctenostomatida; class Phylactolaemata (freshwater), order Phylactolaemata*incertae sedis*; class Stenolaemata, order Cyclostomatida). The order Cheilostomatida is the most diverse.

All bryozoans are clonal and the colonies can take many different forms including encrusting, erect and arborescent forms (McKinney and Jackson 1991). The majority of bryozoan species have a calcium carbonate skeleton, but there are also a number of chitinous and gelatinous species. Colony growth proceeds via the asexual budding of individual units, called zooids, with sexual reproduction producing free-swimming larvae (McKinney and Jackson 1991). Bryozoan species occur in all major marine habitats, from the Polar regions to the tropics, ranging from the intertidal zone to the deep sea. The vast majority of species live attached to a substrate, which may be rocks, biogenic structures (e.g. coral, shells), algae or man-made debris (Hayward and Ryland 1998).

Bryozoans contribute to ecosystem functioning and services through the provision of three-dimensional structure and habitat for other species, and by serving as a food source for other marine species (Bitschofsky et al. 2011; Lidgard 2008). Bryozoans are also recognized for their potential economic importance due to the pharmaceutical and active compounds that are associated with a number of species. (Narkowicz et al. 2002). Several bryozoan species are recognized as invasive and are potentially harmful to native marine species (O’Brien et al. 2013; Yorke and Metaxas 2011). Despite these ecological and economic roles, knowledge on local bryozoan species and faunistic inventories are often lacking or incomplete (Rouse et al. 2014). Such baseline information on species distributions is required to monitor and manage the impact of human activities on the marine environment, and ultimately preserve marine biodiversity (Powney and Isaac 2015).

Scotland lays claim to one of the largest marine resources in Europe with over 9910 km of mainland coastline, 8092 km of island coastline, and an estimated 88,600 km^2^ of territorial seas (Baxter et al. 2011). The west coast of Scotland has numerous exposed islands, high sea cliffs, and fjordic inlets, while the east coast is less variable and dominated by low-lying sedimentary shores. Marine spatial planning has been identified as priority by the Scottish Government (Baxter et al. 2011), and there is a drive towards providing reliable information on species occurrences and distribution. Scotland has historically been the focus of much marine biological research and as such a vast back catalogue of bryozoan records exist (e.g. Norman 1869, Hiscock 1996). These records, however, are often disparate, unreliable and/or difficult to locate. Rouse et al. (2014) analysed records of marine bryozoan from Scotland between 1792 and 2010 to assess spatial and temporal trends in bryozoan diversity. Records were compiled from museum collections, professional/academic surveys, consultancy reports and a citizen science scheme consisting of trained amateurs. Records for which the location was uncertain or not provided, and/or the species seemed likely to be wrong based on its generally accepted distribution (e.g. tropical or Antarctic) were discarded. Other records that had only been documented in Scotland by one source, with an unknown or non-expert identified, were also excluded from the analysis. Approximately 8% of these records were museum collections with associated specimens, 60% from a ten-year expert survey of the British coastline and 16% from the citizen science scheme, with the latter two relying on identification via optical microscopes. The remaining records were compiled from published manuscripts that used a combination of optical and scanning electron microscopy for identification.

Using these records, Rouse et al. (2014) found bryozoan diversity to be higher on the west coast of Scotland than other regions, but this was largely attributed to a sampling bias towards the west coast. The study also highlighted the lack of a bryozoan species list for Scottish waters. The aim of the present study, therefore, is to combine the data collated by Rouse et al. (2014) with recent bryozoan studies in Scotland to provide to a comprehensive species checklist of marine bryozoan species in the region.

## Methods

### Study area

**Figure 1.**
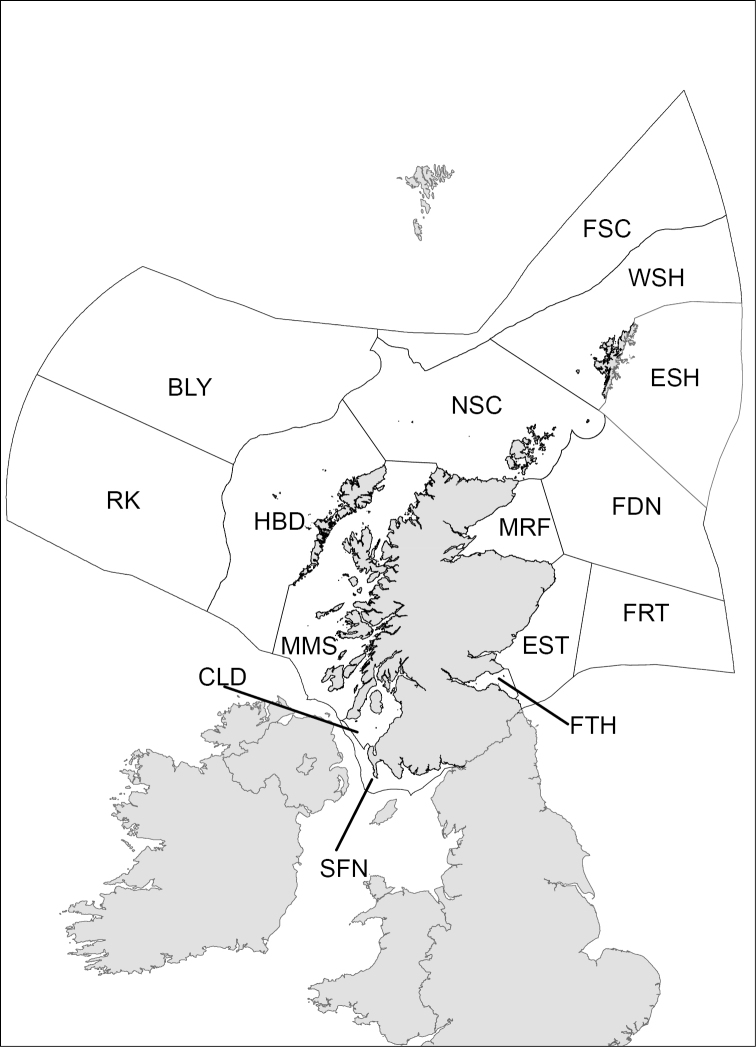
Scottish sea regions. The abbreviations given are used in the checklist. BLY (Bailey), CLD (Clyde), ESH (East Shetland), EST (East Scotland), FDN (Fladen), FRT (Forties), FSC (Faroe-Shetland Channel), FTH (Forth), HBD (Hebrides), MMS (Minches and Malin Sea), MRF (Moray Firth), NSC (North Scotland), RK (Rockall), SFN (Solway Firth and North Channel), WSH (West Shetland).

The Scottish sea region was defined according to the ‘Clean Sea Assessment’ in the Scottish Government’s Marine Atlas (Baxter et al. 2011). The region constitutes 15 sub-regions covering coastal and offshore areas (Figure 1). Previous sub-divisions of the Scottish seas (e.g., the MNCR regions used by Rouse et al. (2014)) are restricted to coastal areas, and as such have not been selected for use in this checklist. There is no a priori reason to expect that the Scottish sea region would have a distinct fauna, however the region does support a greater range of habitat types than the adjoining English Sea area (Baxter et al. 2011). The north of Scotland also represents a transitional area between arctic and boreal species (Boulton et al. 1991).

### Data sources

Historical and contemporary records of bryozoans were obtained from sources including museum collections, literature, and online databases according to the methods of Rouse et al. (2014). These records were supplemented with records from occasional field surveys carried around Scottish harbours and marinas as part of an on-going invasive species survey programme (Collin et al. 2015; Loxton 2014; Nall et al. 2015; Porter et al. 2015; Wasson and De Blauwe 2014). The checklist represents the species known from Scotland up until 2015.

### Organization of the checklist

The checklist is arranged phylogenetically for the higher-level taxa, with the families, genera, and species listed alphabetically. Taxonomy was checked against the World Register of Marine Species (Horton et al. 2016), and names that were currently listed as ‘accepted’ are presented. The number in parentheses immediately to the right of the family name indicates the number of associated taxa, and the abbreviations next to each species specify the sub-region from which records originated (see Figure 1 for definitions of abbreviations). Bryozoan non-indigenous species (NIS) are denoted with an asterisk (*) in the checklist. Individual bryozoan records are provided in the Suppl. material 1.

## Results

Table 1 shows the checklist of marine Bryozoa from the Scottish sea regions. A total of 218 species are included in the list, belonging to 128 different genera from 58 families. The Scottish records represent approximately 3.7% of the total number of bryozoan species known worldwide (n = 5869) (Bock and Gordon 2013). There are representatives from each of the extant marine bryozoan orders (Cyclostomatida, Ctenostomatida, and Cheilostomatida). The most speciose bryozoan families in Scotland were the Calloporidae (13 species) and the Romancheinidae (13 species), which both contain mainly encrusting species.

**Table 1.** Checklist of marine bryozoan fauna occurring in the Scottish sea region. Species denoted with an asterisk (*) indicate those considered to be non-indigenous within Scotland.

**STENOLAEMATA (30)**	
**Order Cyclostomatida (30)**	**Sub-region**
Family Annectocymidae (2)	
*Annectocymamajor* (Johnston, 1847)	ESH, MMS, RK, WSH
*Entalophoroeciadeflexa* (Couch, 1842)	CLD, HBD, MMS, RK
Family Crisiidae (8)	
*Bicrisiaabyssicola* Kluge,1962	HBD, NCS
*Crisiaaculeata* Hassall,1841	CLD, EST, FTH, HBD, MMS, NCS, WSH
*Crisiadenticulata* (Lamarck, 1816)	CLD, EST, FTH, HBD, MMS, NCS, SFN, WSH
*Crisiaeburnea* (Linnaeus, 1758)	CLD, EST, FTH, HBD, MMS, NCS, SFN, WSH
*Crisiaramosa* Harmer, 1891	HBD, MMS
*Crisidiacornuta* (Linnaeus, 1758)	CLD, EST, HBD, MMS, NCS, SFN, WSH
*Crisiellaproducta* (Smitt, 1865)	CLD, WSH
*Filicrisiageniculata* (Milne Edwards, 1838)	CLD, HBD, MMS,
Family Horneridae (1)	
*Horneralichenoides* (Linnaeus, 1758)	ESH, FSC, RK, WSH
Family Lichenoporidae (3)	
*Coronoporatruncata* (Fleming, 1828)	MMS, NCS, RK, WSH
*Disporellahispida* (Fleming, 1828)	CLD, EST, FTH, HBD, MMS, NCS, WSH
*Patinellaverrucaria* (Linnaeus, 1758)	CLD, EST, FTH, MMS, NCS, WSH
Family Oncousoeciidae (2)	
*Oncousoeciadiastoporides* (Norman, 1869)	MRF, WSH
*Oncousoeciadilatans* (Johnston, 1847)	ESH, MMS, WSH
Family Plagioeciidae (2)	
*Diplosolenobelia* (Johnston, 1838)	CLD, ESH, HBD, MMS, WSH
*Plagioeciapatina* (Lamarck, 1816)	CLD, EST, HBD, MMS, NCS, WSH
Family Stigmatoechidae (1)	
*Stigmatoechosviolacea* (M.Sars, 1863)	RK, WSH
Family Stomatoporidae (2)	
*Stomatoporagingrina* Jullien, 1882	RK
*Stomatoporinaincurvata* (Hincks, 1859)	MMS, MRF
Family Terviidae (1)	
*Terviairregularis* (Meneghini, 1844)	RK
Family Tubuliporidae (8)	
*Exidmoneaatlantica* (Forbes in Johnston, 1847)	MMS, RK, WSH
*Tubuliporaaperta* Harmer, 1898	EST, FTH, NCS
*Tubuliporaflabellaris* (O. Fabricius, 1780)	CLD
*Tubuliporaliliacea* (Pallas, 1766)	CLD, EST, FTH, FRT, HBD, MMS, MRF, NCS, RK, WSH
*Tubuliporalobifera* Hastings, 1963	CLD, MMS, MRF, NCS
*Tubuliporapenicillata* (O. Fabricius, 1780)	MMS, MRF
*Tubuliporaphalangea* Couch, 1844	HBD, MMS, NCS, WSH
*Tubuliporaplumosa* Thompson in Harmer, 1898	EST, FTH, MMS, NCS, WSH
**GYMNOLAEMATA (189)**	
**Order Ctenostomatida (27)**	
Family Alcyonidiidae (8)	
*Alcyonidioidesmytili* (Dalyell, 1848)	CLD, EST, FTH, HBD, MMS, NCS, SFN, WSH
*Alcyonidiumalbidum* Alder, 1857	CLD, EST, FTH, MMS, MRF, NCS, WSH
*Alcyonidiumdiaphanum* (Hudson, 1778)	CLD, ESH, EST, FTH, FRT, HBD, MMS, MRF, NCS, RK, SFN, WSH
*Alcyonidiumgelatinosum* (Linnaeus, 1761)	CLD, EST, FTH, FRT, HBD, MMS, MRF, NCS, SFN, WSH
*Alcyonidiumhirsutum* (Fleming, 1828)	CLD, ESH, EST. FTH, HBD, MMS, MRF, NCS, SFN, WSH
*Alcyonidiummamillatum* Alder, 1857	CLD, EST, MMS, MRF, NCS, SFN
*Alcyonidiumparasiticum* (Fleming, 1828)	CLD, EST, FTH, MMS, MRF, NCS, WSH
*Alcyonidiumpolyoum* (Hassall, 1841)	HBD
Family Arachnidiidae (2)	
*Arachnidiumclavatum* Hincks, 1877	CLD, MMS, WSH
*Arachnidiumfibrosum* Hincks, 1880	CLD
Family Buskiidae (1)	
*Buskianitens* Alder, 1857	WSH
Family Farrellidae (1)	
*Farrellarepens* (Farre, 1837)	SFN
Family Flustrellidridae (1)	
*Flustrellidrahispida* (O. Fabricius, 1780)	CLD, EST, FTH, HBD, MMS, MRF, NCS, SFN, WSH
Family Hypophorellidae (1)	
*Hypophorellaexpansa* Ehlers, 1876	CLD
Family Nolellidae (3)	
*Nolelladilatata* (Hincks, 1860)	CLD, FTH, MMS, NCS, WSH
*Nolellapusilla* (Hincks, 1880)	CLD
*Nolellastipata* Gosse, 1855	MMS
Family Spathiporidae (1)	
*Spathiporasertum* Fischer, 1866	WSH
Family Triticellidae (2)	
*Triticellaflava* Dalyell, 1848	CLD, SFN
*Triticellapedicellata* (Alder, 1857)	CLD
Family Vesiculariidae (6)	
*Amathiagracilis* (Leidy, 1855)	CLD, FTH, MMS
*Amathiagracillima* (Hincks, 1877)	MMS
*Amathiaimbricata* (Adams, 1798)	CLD, EST, FTH, HBD, MMS, NCS, SFN
*Amathialendigera* (Linnaeus, 1758)	SFN
*Amathiapustulosa* (Ellis & Solander, 1786)	CLD, MMS, SFN
*Vesiculariaspinosa* (Linnaeus, 1758)	CLD, FTH, MMS, SFN
Family Walkeriidae (1)	
*Walkeriauva* (Linnaeus, 1758)	CLD, ESH, MMS, NCS
**Order Cheilostomatida (160)**	
Family Aeteidae (3)	
*Aeteaanguina* (Linnaeus, 1758)	EST, FTH, HBD, MMS, NCS, WSH
*Aeteasica* (Couch, 1844)	CLD, MMS, NCS
*Aeteatruncata* (Landsborough, 1852)	CLD, MMS, NCS
Family Antroporidae (1)	
*Rosselianarosselii* (Audouin, 1826)	ESH, WSH
Family Beaniidae (1)	
*Beaniamirabilis* Johnston, 1840	EST, MMS, NCS
Family Bitectiporidae (7)	
*Hippoporinapertusa* (Esper, 1796)	CLD, MMS, NCS, SFN, WSH
*Pentaporafascialis* (Pallas, 1766)	HBD, MMS, SFN
*Schizomavellaauriculata* (Hassall, 1842)	MMS, NCS, SFN, WSH
*Schizomavellacornuta* (Heller, 1867)	WSH
*Schizomavelladiscoidea* (Busk, 1859)	NCS, WSH
*Schizomavellahastata* (Hincks, 1862)	WSH
*Schizomavellalinearis* (Hassall, 1841)	CLD, EST, FTH, HBD, MMS, MRF, NCS, SFN, WSH
Family Bryocryptellidae (8)	
*Marguettalorea* (Alder, 1864)	ESH, WSH
*Palmiskeneaskenei* (Ellis & Solander, 1786)	CLD, EST,MMS, MRF, RK, WSH
*Porellaalba* Nordgaard, 1906	EST, MRF, NCS
*Porellacompressa* (J. Sowerby, 1805)	CLD, HBD, MMS, MRF, NCS, RK, WSH
*Porellaconcinna* (Busk, 1854)	CLD, ESH, EST, MMS, MRF, WSH
*Porellalaevis* (Fleming, 1828)	WSH
*Porellaminuta* (Norman, 1868)	MRF, WSH
*Porellastruma* (Norman, 1868)	ESH, WSH
Family Bugulidae (12)	
*Bicellariellaciliata* (Linnaeus, 1758)	CLD, ESH, EST, FTH, HBD, MMS, NCS, WSH
*Bicellarinaalderi* (Busk, 1859)	MMS, NCS, WSH
*Bugulinaavicularia* (Linnaeus, 1758)	CLD, HBD, MMS, NCS, SFN, WSH
*Bugulinacalathus* (Norman, 1868)	MMS
*Bugulinaflabellata* (Thompson in Gray, 1848)	CLD, ESH, EST, FTH, HBD, MMS, MRF, NCS, RK, SFN, WSH
*Bugulinafulva (Ryland, 1960)	MMS, NCS
*Bugulinaturbinata* (Alder, 1857)	CLD, FTH, HBD, MMS, NCS, WSH
*Bugulinasimplex (Hincks, 1886)	CLD, ESH, MMS, NCS
*Bugulaneritina (Linnaeus, 1758)	CLD
*Crisulariaplumosa* (Pallas, 1766)	CLD, EST, FTH, HBD, MMS, NCS, SFN
*Crisulariapurpurotincta* (Norman, 1868)	ESH, EST, FTH, HBD, MMS, NCS, WSH
*Dendrobeaniamurrayana* (Bean in Johnston, 1847)	ESH, MMS, NCS, WSH
Family Calloporidae (13)	
*Alderinaimbellis* (Hincks, 1860)	MMS, NCS, WSH
*Amphiblestrumauritum* (Hincks, 1877)	EST, MMS, NCS, WSH
*Amphiblestrumflemingii* (Busk, 1854)	CLD, EST, FTH, MMS, MRF, NCS, RK, WSH
*Amphiblestrumsolidum* (Packard, 1863)	ESH, MMS, MRF, WSH
*Calloporacraticula* (Alder, 1856)	CLD, MMS, WSH
*Calloporadumerilii* (Audouin, 1826)	MMS, MRF, NCS, SFN, WSH
*Calloporalineata* (Linnaeus, 1767)	CLD, EST, FTH, MMS, MRF, NCS, WSH
*Calloporarylandi* Bobin & Prenant, 1965	EST, FTH, HBD, MMS, NCS
*Cauloramphusspiniferum* (Johnston, 1832)	EST, MMS, NCS, WSH
*Crassimarginatellasolidula* (Hincks, 1860)	EST, WSH
*Megaporaringens* (Busk, 1856)	EST, FSC, WSH
*Ramphonotusminax* (Busk, 1860)	ESH, RK, WSH
*Tegellaunicornis* (Fleming, 1828)	EST, MRF, NCS, WSH
Family Candidae (9)	
*Cabereaellisii* (Fleming, 1814)	NCS, WSH
*Cradoscrupocellariareptans* (Linnaeus, 1758)	CLD, ESH, EST, FTH, HBD, MMS, NCS, SFN, WSH
*Notoplitesharmeri* Ryland, 1963	WSH
*Notoplitesjeffreysii* (Norman, 1863)	ESH, MMS, WSH
*Pomocellariainarmata* (O’Donoghue & O’Donoghue, 1926)	FTH, MMS, WSH
*Scrupocellariascruposa* (Linnaeus, 1758)	CLD, ESH, EST, FTH, HBD, MMS, NCS, SFN, WSH
*Tricellariainopinata d’Hondt & Occhipinti Ambrogi, 1985	CLD, EST, MMS, MRF, NCS
*Tricellariapeachii* (Busk, 1851)	ESH, EST, MRF, NCS, WSH
*Tricellariaternata* (Ellis & Solander, 1786)	ESH, EST, FTH, FRT, HBD, NCS, WSH
Family Cellariidae (4)	
*Cellariafistulosa* (Linnaeus, 1758)	CLD, EST, FTH, HBD, MMS, MRF, NCS, SFN, WSH
*Cellariasalicornioides* Lamouroux, 1816	CLD, MMS, WSH
*Cellariasinuosa* (Hassall, 1840)	CLD, EST, HBD, MMS, SFN, WSH
*Euginomavermiformis* Jullien, 1883	RK
Family Celleporidae (11)	
*Buskeadichotoma* (Hincks, 1862)	CLD, EST, MMS, MRF, WSH
*Buskeanitida* Heller, 1867	CLD, MMS
*Celleporapumicosa* (Pallas, 1766)	CLD, ESH, EST, FTH, FRT, HBD, MMS, MRF, NCS, RK, WSH
*Celleporinacaliciformis* (Lamouroux, 1816)	CLD, ESH, FTH, HBD, MRF, MMS, NCS, WSH
*Celleporinadecipiens* Hayward, 1976	HBD
*Celleporinapygmaea* (Norman, 1868)	FSC, MRF, WSH
*Lageniporalepralioides* (Norman, 1868)	ESH, WSH
*Omalosecosaramulosa* (Linnaeus, 1767)	CLD, ESH, EST, FTH, HBD, MMS, MRF, NCS, WSH
*Palmicellariaelegans* Alder, 1864	WSH
*Turbicelleporaavicularis* (Hincks, 1860)	CLD, EST, FRT, HBD, MMS, MRF
*Turbicelleporaboreale* Hayward & Hansen, 1999	RK
Family Chaperiidae (1)	
*Larnacicuscorniger* (Busk, 1859)	FSC, RK, WSH
Family Chorizoporidae (1)	
*Chorizoporabrongniartii* (Audouin, 1826)	EST, MMS, NCS, SFN, WSH
Family Cribrilinidae (7)	
*Collarinabalzaci* (Audouin, 1826)	CLD, MMS, WSH
*Cribrilinaannulata* (O. Fabricius, 1780)	CLD, EST, FTH, MMS, NCS, WSH
*Cribrilinacryptooecium* Norman, 1903	EST, MMS, MRF, NCS, WSH
*Cribrilinapunctata* (Hassall, 1841)	CLD, EST, FTH, MMS, MRF, NCS, WSH
*Membraniporellanitida* (Johnston, 1838)	CLD, EST, FTH, HBD, MMS, NCS, WSH
*Puellinainnominata* (Couch, 1844)	CLD
*Puellinavenusta* (Canu & Bassler, 1925)	CLD, WSH
Family Cryptosulidae (1)	
*Cryptosulapallasiana* (Moll, 1803)	CLD, MMS, MRF, NCS, WSH
Family Doryporellidae (1)	
*Doryporellinareticulata* (Ryland, 1963)	RK
Family Electridae (7)	
*Aspidelectramelolontha* (Landsborough, 1852)	NCS
*Conopeumreticulum* (Linnaeus, 1767)	CLD, EST, FTH, FRT, MMS, NCS, MRF
*Conopeumseurati* (Canu, 1928)	NCS
*Einhorniacrustulenta* (Pallas, 1766)	NCS
*Electramonostachys* (Busk, 1854)	MMS, NCS, SFN
*Electrapilosa* (Linnaeus, 1767)	CLD, ESH, EST, FTH, HBD, MMS, MRF, NCS, RK, SFN, WSH
*Pyriporacatenularia* (Fleming, 1828)	CLD, FRT, MMS, NCS, SFN, WSH
Family Escharinidae (5)	
*Escharinaalderi* (Busk, 1856)	FSC, MMS, RK, WSH
*Escharinadutertreihaywardi* Zabala, Maluquer & Harmelin, 1993	FSC, WSH
*Escharinajohnstoni* (Quelch, 1884)	CLD, MMS
*Herentiahyndmanni* (Johnston, 1847)	NCS, WSH
*Phaeostachysspinifera* (Johnston, 1847)	FTH, MMS, NCS, WSH
Family Eucrateidae (1)	
*Eucratealoricata* (Linnaeus, 1758)	CLD, ESH, EST, FTH, HBD, MMS, MRF, NCS, SFN, WSH
Family Exechonellidae (1)	
*Anarthroporamonodon* (Busk, 1860)	FSC, WSH
Family Exochellidae (2)	
*Escharoidescoccinea* (Abildgaard, 1806)	CLD, EST, FTH, HBD, MMS, MRF, NCS, WSH
*Escharoidesmamillata* (Wood, 1844)	EST, MMS, NCS, WSH
Family Flustridae (7)	
*Carbaseacarbasea* (Ellis & Solander, 1786)	EST, FTH, HBD, WSH
*Chartellabarleei* (Busk, 1860)	ESH, NCS, WSH
*Chartellapapyracea* (Ellis & Solander, 1786)	CLD, HBD, MMS
*Flustrafoliacea* (Linnaeus, 1758)	CLD, ESH, EST, FTH, FRT, HBD, MMS, MRF, NCS, SFN, WSH
*Hincksinaflustroides* (Hincks, 1877)	HBD
*Sarsiflustraabyssicola* (Sars G.O., 1872)	WSH
*Securiflustrasecurifrons* (Pallas, 1766)	CLD, ESH, EST, FTH, FRT, HBD, MMS, MRF, NCS, SFN, WSH
Family Haplopomidae (4)	
*Haplopomagraniferum* (Johnston, 1847)	CLD, FTH, NCS, WSH
*Haplopomaimpressum* (Audouin, 1826)	CLD, MMS, NCS, WSH
*Haplopomaplanum* Ryland, 1963	ESH, WSH
*Haplopomasciaphilum* Silén & Harmelin, 1976	HBD
Family Hippoporidridae (2)	
*Hippoporellahippopus* (Smitt, 1867)	MRF
*Hippoporidralusitania* Taylor & Cook, 1981	WSH
Family Hippothoidae (4)	
*Celleporellahyalina* (Linnaeus, 1767)	CLD, EST, FTH, HBD, MMS, MRF, NCS, WSH
*Haplotaclavata* (Hincks, 1857)	CLD
*Hippothoadivaricata* Lamouroux, 1821	CLD, EST, NCS
*Hippothoaflagellum* Manzoni, 1870	CLD, MMS, NCS
Family Lacernidae (1)	
*Cylindroporellatubulosa* (Norman, 1868)	HBD, MRF, NCS, WSH
Family Membraniporidae (1)	
*Membraniporamembranacea* (Linnaeus, 1767)	ESH, EST, FTH, HBD, MMS, MRF, NCS, RK, SFN, WSH
Family Microporellidae (3)	
*Fenestrulinadelicia* Winston, Hayward & Craig, 2000	CLD, WSH
*Fenestrulinamalusii* (Audouin, 1826)	CLD, EST, HBD, MMS, MRF, NCS, SFN, WSH
*Microporellaciliata* (Pallas, 1766)	CLD, EST, FTH, MMS, NCS, SFN, WSH
Family Microporidae (3)	
*Microporacoriacea* (Johnston, 1847)	CLD
*Microporanormani* Levinsen, 1909	WSH
*Molliamultijuncta* (Waters, 1879)	WSH
Family Phidoloporidae (5)	
*Reteporellabeaniana* (King, 1846)	MMS, NCS, RK, WSH
*Reteporellaincognita* Hayward & Ryland, 1996	RK, WSH
*Reteporellawatersi* (Nordgaard, 1907)	WSH
*Rhynchozoonbispinosum* (Johnston, 1847)	WSH
*Schizothecafissa* (Busk, 1856)	MMS
Family Romancheinidae (13)	
*Arctonulaarctica* (M. Sars, 1851)	EST, WSH
*Escharellaabyssicola* (Norman, 1869)	FSC, WSH
*Escharellaimmersa* (Fleming, 1828)	CLD, EST, MMS, MRF, NCS, WSH
*Escharellalabiosa* (Busk, 1856)	HBD, MMS
*Escharellalaqueata* (Norman, 1864)	MMS, WSH
*Escharellaoctodentata* (Hincks, 1880)	FSC, RK, WSH
*Escharellavariolosa* (Johnston, 1838)	CLD, EST, MMS, MRF, WSH
*Escharellaventricosa* (Hassall, 1842)	CLD, EST, FTH, MMS, MRF, NCS, WSH
*Hemicycloporapolita* (Norman, 1864)	ESH, MMS, WSH
*Neolageniporacollaris* (Norman, 1867)	MMS, MRF, NCS, WSH
*Neolageniporaeximia* (Hincks, 1860)	WSH
*Ragionularosacea* (Busk, 1856)	CLD, NCS, WSH
*Temachiamicrostoma* (Norman, 1864)	ESH, WSH
Family Schizoporellidae (6)	
*Schizoporellacornualis* Hayward & Ryland, 1995	MMS
*Schizoporelladunkeri* (Reuss, 1848)	MMS, NCS, WSH
*Schizoporellajaponica Ortmann, 1890	CLD, ESH, EST, MMS, MRF, NCS, WSH
*Schizoporellapatula* Hayward & Ryland, 1995	ESH, FSC, NCS, WSH
*Schizoporellaumbonata* O’Donoghue & O’Donoghue, 1926	WSH
*Schizoporellaunicornis* (Johnston in Wood, 1844)	CLD, HBD, MMS, MRF, NCS, WSH
Family Scrupariidae (2)	
*Scrupariaambigua* (d’Orbigny, 1841)	EST, HBD
*Scrupariachelata* (Linnaeus, 1758)	CLD, EST, FTH, HBD, MMS, NCS, WSH
Family Setosellidae (1)	
*Setosellavulnerata* (Busk, 1860)	ESH, WSH
Family Smittinidae (8)	
*Parasmittinatrispinosa* (Johnston, 1838)	CLD, ESH, EST, FTH, HBD, MMS, MRF, NCS, RK, SFN, WSH
*Phylactellalabrosa* (Busk, 1854)	MRF, NCS, WSH
*Pseudoflustravirgula* Hayward, 1994	FSC
*Smittinabella* (Busk, 1860)	CLD, EST, WSH
*Smittinacrystallina* (Norman, 1867)	MMS, MRF, NCS, WSH
*Smittoideaamplissima* Hayward, 1979	WSH
*Smittoideamarmorea* (Hincks, 1877)	EST, FTH, MMS, NCS, WSH
*Smittoideareticulata* (MacGillivray, 1842)	CLD, EST, FTH, MMS, MRF, NCS, WSH
Family Stomachetosellidae (3)	
*Stomachetosellanormani* Hayward, 1994	WSH
*Stomacrustulacruenta* (Busk, 1854)	CLD, ESH, WSH
*Stomacrustulasinuosa* (Busk, 1860)	CLD, MMS, WSH
Family Tessaradomidae (1)	
*Tessaradomaboreale* (Busk, 1860)	HBD, RK, WSH
Family Umbonulidae (1)	
*Oshurkovialittoralis* (Hastings, 1944)	CLD, ESH, EST, FTH, HBD, MMS, MRF, NCS SFN, WSH

Six NIS were identified as part of the Scottish fauna. These were *Bugulinafulva* (Ryland, 1960), *Bugulinasimplex* (Hincks, 1886), *Bugulaneritina* (Linnaeus, 1758), *Tricellariainopinata* d’Hondt & Occhipinti Ambrogi, 1985, *Fenestrulinadelicia* Winston, Hayward & Craig, 2000, *Schizoporellajaponica* Ortmann, 1890. The Clyde sub-region contained the greatest number of NIS (all except *B.fulva*).

## Discussion

The Scottish sea regions contain 218 bryozoan species with representatives from each of the extant bryozoan orders. Based on the checklist, it can be concluded that Scotland has fewer bryozoan species than New Zealand (n = 953), Australia (n = 886), and the Mediterranean (n = 556) (Gordon 1999; Gordon et al. 2010; Rosso and Di Martino 2016). Given Scotland’s location within a single biogeographical region, this relative lack of species is as expected (Baxter et al. 2011). When coastline length is accounted for, Scotland has approximately half the number of species per km (0.01) as Australia (0.02 species/km) and approximately six times fewer than New Zealand (0.06 species/km). The proportion of ctenostomes in Scotland (12% of total species) is greater than the global average (~5%) (Bock and Gordon 2013), and greater than the proportion of ctenostomes reported from New Zealand (5%), Australia (4%), Argentina (4%) and the Mediterranean (10%) (Gappa 2000; Gordon 1999; Rosso and Di Martino 2016). Only the bryozoan fauna of Brazil has a greater percentage (26.2%) of ctenostomes. Previously, higher incidences of ctenostomes (and/or cyclostomes) have been attributed to the results of focused taxonomic efforts in certain regions (Gappa 2000; Rosso 2003). Rosso and Di Martino (2016), however, suggested that the abundance of ctenostomes in the Mediterranean could also reflect the availability of high-energy algal and seagrass dominated habitats, for which the flexible uncalcified ctenostome colony forms are well adapted to exploit. Scotland, and the Scottish west coast in particular, has a high abundance and diversity of algae and algal dominated habitats (Smale et al. 2013), which may explain the high number of ctenostomes found in the study region.

As with other benthic marine invertebrates in Scotland, the bryozoan fauna includes NIS (Nall et al. 2015). The presence of all but one NIS within the Clyde Sea region most likely represents the fact that the area is both a well-studied region and the location of a significant number of ports. As global shipping and aquaculture increase, along with climate change, it is expected that the number of invasive or non-indigenous bryozoans in the Scottish sea regions will increase in the future (Stretaris et al. 2005).

The estimate of bryozoan species number in Scotland, presented here, is likely to be conservative, since much of the offshore shelf areas and seamounts have not been fully explored. Estimates of the global number of bryozoan species yet to be discovered range from 2800–5200 (Appeltans et al. 2012). Given that the Scottish bryozoan fauna currently constitutes 3.7% of global bryozoan species richness, and assuming that this proportion will remain constant, it could be expected that there are approximately 104–192 bryozoan species in Scotland yet to be discovered.

## Supplementary materials

Supplementary material 1 Scottish bryozoan records Data type: occurence This is an open access article distributed under the terms of the Creative Commons Attribution License (CC BY 4.0), which permits unrestricted use, distribution, and reproduction in any medium, provided the original author and source are credited.Sally Rouse, Jennifer Loxton, Mary E. Spencer Jones, Joanne S. Porter
